# Supplementary material for: Course of illness and outcomes in older COVID-19 patients treated with HFNC: a retrospective analysis
Source: Aging (Albany NY). 2021 Jun 28;13(12):15801–14. doi: 10.18632/aging.203224 (PMC8266360; doi:10.18632/aging.203224)
Supplement: Supplementary Table 1 [file aging-13-203224-s001.pdf]

SUPPLEMENTARY TABLE

Supplemental Table 1. Multivariate logistic regression analysis of factors related to older COVID-19 patients.

Variable	B	SE	Wald	*P*-value	Odds rate	95% CI	
						low	up
HR	0.016	0.28	4.863	0.027	0.941	0.891	0.993
SpO_2	0.245	0.099	4.057	0.014	1.277	1.051	1.552
Lactate concentration	1.127	0.413	7.431	0.006	3.087	1.373	6.941
PaO_2/FiO_2 HFNC onset	1.036	0.015	5.454	0.020	2.036	2.006	2.068
APTT	0.116	0.059	3.824	0.051	1.123	1.000	1.262

non-survival.

B, regression coefficient; SE, Standard Deviation; 95%CI, 95% Confidence interval. SpO_2= pulse oxygen saturation, FiO_2=fraction of inspired oxygen, PaO_2=partial pressure of oxygen, HFNC=high flow nasal cannula, SOFA score = sequential organ failure assessment score. APECHE II scores = acute physiology and chronic health evaluation II score, APTT=Activated partial thromboplastin time.
